# An instrument for quality assurance in work capacity evaluation: development, evaluation, and inter-rater reliability

**DOI:** 10.1186/s12913-019-4387-4

**Published:** 2019-08-09

**Authors:** André Strahl, Christian Gerlich, Georg W. Alpers, Jörg Gehrke, Annette Müller-Garnn, Heiner Vogel

**Affiliations:** 1 Department of Medical Psychology, Medical Sociology, and Rehabilitation Sciences, University of Wuerzburg, Klinikstr. 3, 97070 Wuerzburg, Germany; 2 Department of Orthopaedics, University Medical Center Hamburg-Eppendorf, Martinistr. 52, 20246 Hamburg, Germany; 3 Department of Psychology, School of Social Sciences, University of Mannheim, 68131 Mannheim, Germany; 4 Department of Social Medicine, German Statutory Pension Insurance, Ruhrstr. 2, 10709 Berlin, Germany

**Keywords:** Work capacity evaluation, Insurance medicine, Quality assurance, Peer review, Reliability

## Abstract

**Background:**

Employees insured in pension insurance, who are incapable of working due to ill health, are entitled to a disability pension. To assess whether an individual meets the medical requirements to be considered as disabled, a work capacity evaluation is conducted. However, there are no official guidelines on how to perform an external quality assurance for this evaluation process. Furthermore, the quality of medical reports in the field of insurance medicine can vary substantially, and systematic evaluations are scarce. Reliability studies using peer review have repeatedly shown insufficient ability to distinguish between high, moderate and low quality. Considering literature recommendations, we developed an instrument to examine the quality of medical experts’ reports.

**Methods:**

The peer review manual developed contains six quality domains (formal structure, clarity, transparency, completeness, medical-scientific principles, and efficiency) comprising 22 items. In addition, a superordinate criterion (survey confirmability) rank the overall quality and usefulness of a report. This criterion evaluates problems of inner logic and reasoning. Development of the manual was assisted by experienced physicians in a pre-test. We examined the observable variance in peer judgements and reliability as the most important outcome criteria. To evaluate inter-rater reliability, 20 anonymous experts’ reports detailing the work capacity evaluation were reviewed by 19 trained raters (peers). Percentage agreement and Kendall’s W, a reliability measure of concordance between two or more peers, were calculated. A total of 325 reviews were conducted.

**Results:**

Agreement of peer judgements with respect to the superordinate criterion ranged from 29.2 to 87.5%. Kendall’s W for the quality domain items varied greatly, ranging from 0.09 to 0.88. With respect to the superordinate criterion, Kendall’s W was 0.39, which indicates fair agreement. The results of the percentage agreement revealed systemic peer preferences for certain deficit scale categories.

**Conclusion:**

The superordinate criterion was not sufficiently reliable. However, in comparison to other reliability studies, this criterion showed an equivalent reliability value. This report aims to encourage further efforts to improve evaluation instruments. To reduce disagreement between peer judgments, we propose the revision of the peer review instrument and the development and implementation of a standardized rater training to improve reliability.

**Electronic supplementary material:**

The online version of this article (10.1186/s12913-019-4387-4) contains supplementary material, which is available to authorized users.

## Background

To determine whether employees are eligible for a disability allowance following injury or illness, a work capacity evaluation is conducted. This generally involves a physical examination by a social-medical physician. While it is clearly important to verify the accuracy of these evaluations, there are no official guidelines on how to perform an external quality assurance for this evaluation process. The examination is concluded with a medical experts’ report. The quality of these reports can vary substantially. Quality deficits may arise as a result of systematic bias or random error. Systematic bias refers to non-random tendency within the medical assessment to obtain certain results, while random errors describe random variations and variability that influence examination situations. For instance, if patients provide unreliable medical information, this endangers the quality of medical reports and the related work capacity judgment. The judgement is inevitably influenced by physicians’ individual characteristics, experiences, and ideologies [1–3]. Consequently, agreement between two independent medical experts assessing the same patient is rare [4–6]. Social judgement theory has been used to explore and analyze differences in expert judgements, and has referred to, for example, the lens model [7]. According to this model, experts acting within the same context and with access to identical medical information may have different judgments due to systematic differences in how information is processed. This observable phenomenon is attributable to variety of factors: different organisational principles for how to combine information in an appropriate way, different weights for specific pieces of information, and differences in understanding of the importance of information for the judgment. This leads to differences in diagnosis and treatment across physicians [8, 9]. Despite this variability and the susceptibility to errors, medical examination and decision-making are always based on a physician’s judgement. It is therefore important to develop a tool that assesses the external quality assurance for work capacity evaluation.

This circumstance also applies when verifying social security insurance claims. Social security, especially statutory pension funds, entitles an insured person to receive disability pension in case of being incapable of working due to ill health [10, 11]. If specific legal and medical requirements are met, disability pension is allocated to compensate the permanent financial loss. Insured persons at risk of reduced earning capacity are subject to a medical examination in the context of a work capacity evaluation whenever their medical records yield insufficient information [12, 13]. Medical examinations employ the International Classification of Functioning, Disability and Health (ICF), which includes the individual evaluations of work-related somatic, psychological, and social conditions in the realm of work. Rather than simply focusing on diagnosis or disorder itself, the ICF encompasses functional aspects of diseases affecting the daily work life [14]. Finally, the medical experts’ reports are used as basis for subsequent socio-legal decision process. To avoid wrong decisions, these reports should therefore be reliable and valid.

### Quality assurance in work capacity evaluation

In Germany, more than 800 employed physicians and additional 1000 external physicians employed on a fee basis evaluate the work capacity on behalf of 16 departments of the German Statutory Pension Insurance. To avoid systematic bias and random errors, the German Statutory Pension Insurance has developed several quality-assurance measures, including guidelines on how to evaluate the work capacity in terms of relevant chronic diseases, key instructions on how to write and structure a medical experts’ report, and quality circles that foster compliance with these formalities [15]. However, an external quality assurance for medical experts’ reports on work capacity evaluations is missing. In principle, formal and content-related quality of reports can be assessed as a characteristic of outcome quality. Thus, we developed a quality assurance method based on a standardized peer review manual to examine the quality of medical experts’ reports. Because the quality assurance programme should be obligatory for all 16 departments of the German Pension Insurance, the peer review was developed in consultation with all institutions.

### Quality assurance with peer review

Peer review is an established method of external quality assurance in health services [4, 16–18]. Inter-rater reliability is the most important criterion to guarantee fair quality comparison between two or more institutions [4]. It describes the degree to which two or more peers are able to differentiate among the quality of reports (e.g. high, moderate, low quality) under similar assessment conditions (refer to [19]). High inter-rater reliability ensures that an assessment is not dependent on any specific reviewer [20]. The large variability of inter-rater reliability may depend on the type of review objects, as well as on the experience and training of peers. Structured implicit reviews employ standardized data sources and test questions along with pre-defined quality criteria that should ensure adequate to good reliability [21]. Empirical studies, however, did scarcely confirm these assumption [22–29]. In a meta-analysis on reliability of peer assessments, Goldman reported an average weighted Kappa of 0.31 [4], while a systematic review by Lilford and colleagues reported a wide range of inter-rater reliability (Cohen’s Kappa) from 0.32 to 0.70 [30]. To improve high inter-rater reliability, a sound peer review instrument and an associated peer training has been proposed [31]. Furthermore, reliability may also be improved by statistical modifications, such as calculating reliability coefficients that take alternative forms of non-agreement into account [32, 33]. However, the fact remains that peer reviews based on medical records rarely exceed common cut-off criteria for good or acceptable reliability (e.g., [34, 35]).

In view of these empirical findings, the primary objective of this study was to develop and evaluate a peer review instrument to measure the quality of work capacity evaluation using uniform criteria for assessing quality. To do this, we (1) developed a manual comprising a well-defined catalogue of test items, which can be used in peer review and train users of the instrument to (2) evaluate the inter-rater reliability. In addition, individual differences in the peer judgment (peer bias) have been investigated.

## Methods

### Development of the peer review manual

In a conception and pre-test phase, the peer review manual was developed based on preliminary work from board resolutions by the German Statutory Pension Insurance [36], and was put into practice in close coordination with their representatives. A catalogue of proposed quality domains, test items, and an associated evaluation system were examined and included in a peer review manual.

### Conception phase

During the conception phase, predefined quality domains, test items, and the evaluation system were subjected to a critical review by the investigators in charge. After examination of contents, test items were reformulated to enhance their clarity and understandability. The definition of the ordinal rating scale was discussed with the German Pension Insurance. Due to overlapping content, some test questions were removed, and their subject matter was incorporated into other items. In cooperation with the socio-medical service of the German Pension Insurance, a grading system was developed, which was further reviewed and edited in four revision rounds with the German Statutory Pension Insurance.

### Structure of the peer review manual

The final peer review manual encompasses a catalogue of 23 items addressing six subsidiary quality domains as well as one superordinate criterion that measures the confirmability of medical experts’ reports (Table 1). The outcome quality of reports should be assessed in each of these quality domains (formal structure, clarity, transparency, completeness, medical-scientific principles, efficiency). If deficiencies occur in these domains, a report is not fundamentally considered unusable.

**Table 1:** Items and reliability of the revised version of the peer review-manual for quality assurance in work capacity evaluation (n = 325)

Item no.	Test question	Percentage agreement	r_w (n = 325)_
Quality domain: formal structure			
A.1	To what extent does the report structure complied with the requirements?	67.5%	0.19
A.2	To what extent is the unified set form, consisting of cover and back banner page, used?	82.5%	0.89
Quality domain: clarity			
B.1	To what extent is the linguistic expression correct and unambiguous?	69.7%	0.27
B.2	To what extent are technical terms and abbreviations that are essential for the understanding of the report explained?	50.5%	0.36
B.3	To what extent is the specific social medicine terminology applied correctly?	68.1%	0.16
B.4^a^	To what extent are socio-legal implementations / conclusions omitted?	80.6%	0.29
Quality domain: transparency			
C.1	To what extent is the origin of medical information described?	53.8%	0.27
C.2	To what extent does the report illustrated by which processes, methods and tools the medical results are collected?	66.0%	0.37
C.3	To what extent does the report illustrated which measured values, reference ranges and graduations are basis of the medical assessment?	57.9%	0.30
Quality domain: completeness			
D.1	To what extent is the medical anamnesis depicted completely?	48.9%	0.27
D.2^a^	To what extent are medical findings documented to answer the social medicine report questions?	64.6%	0.31
D.3	To what extent are ICD diagnosis illustrated with their functional limitations?	49.2%	0.45
D.4^a^	To what extent are complaints, diseases and functional limitations expressed by the insured included in the discharged summary?	46.2%	0.15
D.5^a^	To what extent are medical findings included in the discharged summary?	52.2%	0.22
D.6^a^	To what extent are functional limitations in relation to performance in working life evaluated?	52.7%	0.09
D.7	To what extent are statements on previous therapy and future therapeutic options given?	39.8%	0.36
D.8	To what extent are substantial differences in the work capacity evaluation compared to earlier medical reports explained?	79.9%	0.25
D.9	To what extent are all social medicine report questions fully answered?	43.9%	0.17
Quality domain: medical-scientific principles			
E.1	To what extent is the widely accepted state of medical knowledge applied?	88.6%	0.27
E.2	To what extent is the existing literature for work capacity evaluation of the German Pension applied?	65.6%	0.24
Quality domain: efficiency			
F.1	To what extent is the diagnostic investigation appropriate and necessary?	72.5%	0.20
F.2	To what extent is the diagnostic investigation sufficient?	57.3%	0.22
Superordinate criterion: experts’ report confirmability			
Evaluate the confirmability of the medical report on the basis of the argumentation used.		47.3%	0.39

Quality domains: four-point rating scale (no deficiencies, mild deficiencies, clear deficiencies, serious deficiencies); superordinate criterion: three-point rating scale (no argument interruptions; argument interruptions that can be bridged by the assessing peer; argument interruptions that cannot be bridged by the assessing peer); r_w_ = Kendall’s coefficient of concordance W

^a^ items has been removed from the manual in agreement with the German Statutory Pension Insurance after this present inter-rater reliability study was completed

Each quality domain was operationalized by a pre-defined set of test items. The number of items as well as the scope of surveyed facts varied for each quality domain. To ensure uniform application of the peer review manual, items were designed using a standardized structure. Namely, each item was accompanied by detailed instructions on the rateable quality deficiencies with examples. Peers rated each item using a four-point ordinal rating scale (no deficiencies, mild deficiencies, clear deficiencies, or serious deficiencies). Furthermore, these ordinal judgment possibilities were guided by pre-defined item-specific anchor definitions that describe possible quality restrictions. It is possible to distinguish between four different types of anchor definition:

1. Grading based on quantitative increase: categories of deficiencies are defined by an ascending characteristic comparable with an ordinal scale (e.g., item B.2: explanation of a technical term is missing once/several times/most of the time).
2. Grading based on quantitative content sensitive increase: the rating quality deficiencies are defined based on a content-wise increasing characteristic (e. g., item B.3: “there are failures, but this results in no incomprehensible conclusions / misleading conclusions / inevitably wrong conclusions”).
3. Grading based on different content: there is no quantitative ranking of deficiencies. Instead, deficiency categories are defined according to different individual aspects comparable with a nominal scale (e.g., item D.9: “there are no statements concerning the need for rehabilitation / the beginning and duration of disease / the qualitative work performance”).
4. Grading based on socio-medical relevance: categories of deficiencies are differentiated according to socio-medical relevance of the deposited criterion (e.g., item C.3: “the exact value for one socio-medically measurement which is not decisive for the work capacity evaluation is missing / the exact values for more than one socio-medically measurements which is not decisive for the work capacity evaluation are missing / the exact value for at least one socio-medically measurement which is decisive for the work capacity evaluation is missing”).

Additional file 1: Table S1 shows an example of the structure of a complete review item.

The superordinate criterion of confirmability evaluates fundamental disruptions in a medical report’s line of argument. The peers should evaluate the meaningful combination of individual assessment steps, e.g., by bridging information from anamnesis and medical findings to socio-medical epicrisis (discharged summary) and, in the following, from epicrisis to work capacity. The argumentation line also includes a comprehensive description of functional limitations and disabilities. By assessing this criterion, peers make an overall judgment on the verifiability of reports. Peers evaluate the gaps in the argumentation on a three-point ordinal scale (no argument interruptions; argument interruptions that can be bridged by the assessing peer; argument interruptions that cannot be bridged by the assessing peer). If there were flaws in the argumentation line, applicability of the report could be questioned according to peer review. In addition to the superordinate criterion 22 items delineate the six subsidiary quality domains listed in Table 1.

### Pre-test phase

After construction, the manual was pre-tested to investigate its practicality, and to identify any possible variance or errors. Twelve medical experts (peers) took part in the pre-test, representing the specialist fields surgery (*n* = 2), orthopaedics (*n* = 1), internal medicine (*n* = 3), general practice (n = 3), and neurology-psychiatry (n = 3). The selection of peers was coordinated by the Social Medicine Department of the German Statutory Pension Insurance. To ensure an unbiased assessment, selected peers were not involved in development process to date, and should not yet have knowledge of the manual (quality domains, items, evaluation system). The peers did not receive any training on how to use the manual yet. Conceptually, the manual and its items should be formulated in a standardised way and be self-explanatory to the extent that no additional peer training should be required.

Out of a pool of 24 medical expert’s reports, every peer assessed six reports by following the manual. Each report was evaluated by three peers resulting in 72 reviews in total. Structure, scope, design, clarity, and understandability of the test items were rated from very good to very poor on a six-point rating scale. Whenever judging a test item, possible difficulties were recorded by the investigating peer. In addition, peers recorded the time spent for reviewing a report. Inter-rater reliability for the superordinate criterion was exploratively computed to obtain a first indication of the quality of peer review. Fleiss’ Kappa was calculated to assess the agreement of judgments. This coefficient is used when the same objects are judged by a fixed number of raters [20, 37].

### Peer review process and inter-rater reliability

Inter-rater reliability was assessed based on a revised version of the manual (see Table 1) once the pre-test was completed. Overall, 19 peers who were affiliated with 12 participating regional pension insurance institutions took part in the evaluation. All peers were medical physicians that volunteered for the study. Peers had considerable experience in social medical services in writing reports themselves or evaluating reports from external experts. They were specialized in surgery (*n* = 3), orthopaedics (n = 3), internal medicine/general practice (*n* = 9), and neurology/psychiatry (*n* = 4). None of the participants had previously been involved in external quality assurance.

All peers attended a two-day peer training. Under guidance they learned how to use the manual by reviewing two selected reports. Individual judgements were discussed in the light of predefined expert judgements according to the manual. These predefined judgements were carried out by a medical expert’s panel in the field of social medicine from the German Pension Insurance. Review and discussion took place in the setting of two small groups of approximately ten peers supervised by these medical experts. During training, all peers assessed the same reports. The task of the moderators, who were experienced in social medicine, was to calibrate all peers to facilitate uniform application of the test items. The main objective of working in small groups was to minimize the peers’ scope for interpretation and to follow the rules of the manual exclusively when assessing experts’ reports. After training, the peers practiced individually applying the manual on three test reports. Following these reports, all peers were contacted by telephone and interviewed about their experiences and problems. To evaluate the success of the training, consensus was defined as the percentage of consistent assessments in the most frequently selected category of deficiencies (mode). After completing the training, a five-month review period followed.

### Review phase

In this study, 20 anonymous experts’ reports, detailing the work capacity evaluation of disability pension claimants, were simultaneously assessed by all peers to determine inter-rater reliability and individual differences in peer judgments. In addition to these 20 reports, 240 experts’ reports have been evaluated by two peers each to characterize the range of different reliability coefficients. The results of this analysis are published elsewhere [38]. The reports were randomly selected and addressed medical problems from the three major medical indications: surgery/orthopaedics, internal medicine/general practice, and neurology/psychiatry. The reports must have been drawn up within the last 12 months. Further, the claimant should not have received a medical rehabilitation one year before the work capacity evaluation. Reports differ in length depending on individual case and major indication. The evaluation included medical experts’ reports from employed physicians as well as external experts, who were required to comply with the published guidelines for writing reports [39].

Peer review was designed as an inter-specialist procedure in which rater evaluate reports, independent of their medical discipline. Concordance was measured with percentage agreement and Kendall’s coefficient of concordance W (r_w_). This coefficient can be calculated for two or more judges providing ordinal data. Furthermore, non-agreement is considered in a graduated way [20]. According to the interpretation guidelines by Altman [34] and Landis and Koch [35], reliability values from 0.00 to 0.20 indicate slight, 0.21 to 0.40 fair, 0.41 to 0.60 moderate and 0.61 to 0.80 substantial agreement.

All peers were informed about the study, received project information, and gave written consent to participate. All study participants who took part in the pre-test and in the evaluation of the inter-rater reliability operated with anonymous medical experts’ reports. As the peer review was performed with an online survey, only anonymized data were processed and evaluated by the researchers in charge. By analysing only anonymous data (no code list, no personal reference possible), an ethic approval from an ethic review board was not required. This approach complies with national ethical guidelines [40].

## Results

### Pre-test of the peer review manual

The mean duration to review one medical report was 57 (SD 30.2) minutes. Assessment of reports from the medical specialist field of internal medicine/general practice took the longest (62 (SD 24.4) minutes), followed by orthopaedics/surgery (60 (SD 52.7) minutes) and neurology/psychiatry (50 (SD 19.3) minutes). A comparison between individual reviewers showed significant differences in length of time needed to perform one review, with an average processing time ranging from 27 to 133 min.

Assessing difficulties applying the manual, peers indicated in 10% of the 72 reviews to had issues applying the superordinate criterion. The other 22 test items showed a significant scattering from 3% (item A.2) to 29% (item E.2). Most problems were reported with test items from the two quality domains medical-scientific principles and efficiency. The overall structure of the manual was rated with a score of 1.8 (1 = very good, 6 = insufficient). Understandability of the items was rated most critically, with a mean of 3.2. Table 2 provides results for each specialist fields.

**Table 2:** Descriptive results for the formal review of the manual (*n* = 11; missing = 1)

Criterion	M	Range	M	M	M
How do you judge …			IM/GP	O/S	N/P
… the structure of the manual	1.8	1–3	1.8	2.3	1.3
… the extent of the manual	2.6	1–5	1.8	3.8	2.3
… the layout of the manual	2.3	1–4	1.8	2.3	3.0
… the clarity of the quality domains and their test items	2.1	1–3	1.8	2.3	2.3
… the understandability of the manuals‘introduction	2.1	1–4	1.8	2.5	3.0
… the understandability of the test items	3.2	2–5	2.5	4.0	2.3
… the manual in total	2.4	1–3	2.3	2.5	2.4

six-point rating scale (1 = very good; 6 = insufficient). *M* Mean, *IM/GP* Internal medicine/general practitioner, *O/S* Orthopaedic/surgery, *N/P* Neurology/psychiatry

The pre-test results revealed a need for revision of the manual. Only half of the participating peers judged the practical realisation of the test items to be adequate. The revision was performed benefiting from 215 annotations that were made by 12 physicians during the pre-test. Test item A.2 was the only question that was handled without any further problems or suggestions for improvement. Peers’ annotations focused on items per se, items descriptions or categories of the four-point ordinal rating scale. In most cases, the aim was to sharpen and clarify individual wording because items were ambiguous. For example, item D.3 (“To what extent are ICD diagnoses illustrated?”) was appended to include “… with their functional limitations” (see Table 1). To simplify the items, it was proposed that specific report sections should be marked to which the items refer. In case of item D.3, the item should only refer to diagnoses section and not to other sections of the report.

Other annotations described what should not be subject to peer review. Item B.1 (“To what extent is the linguistic expression correct and unambiguous?”) should only be evaluated as long as it did not extend to a critique of an expert’s writing style. In some cases, all deficiency categories were rewritten. This structural change led to the anchor definition type, in which the ordinal grading was based on socio-medical relevance (e.g., items C.1 to C.3) to take into account the impact on work capacity evaluation. Four test items were fundamentally reformulated, and several text passages were modified that concerned the detailed item instructions and the ordinal deficit categories. All amendments were discussed in several feedback rounds with the German Statutory Pension Insurance and experts in the field and were finally approved.

Exploratory inter-rater reliability analysis with regard to the superordinate criterion was calculated using Fleiss’ Kappa. In the group of internal medicine/general practice we found a percentage agreement of 41% between all rater pairs. These values, however, did not differ significantly from the expected random match rate of 34% (κ_m_ = 0.11, *p* > 0.05). Agreement of peers with neurology/psychiatry expertise amounted 33%, and was therefore located within random range (κ_m_ = − 0.04, p > 0.05). Orthopaedics/surgery peers achieved moderate agreement. The observed peer agreement of 67% was significantly higher than the random expected agreement of 36% (κ_m_ = 0.48, *p* < 0.05).

### Training results

After peer training, all participating peers assessed three reports according to the revised peer review manual. Data from 57 reviews (three reports by each of the 19 peers) were collected. A verifiable review was lost during digital transmission. Digital access was not possible for this review, which was excluded from further analysis. Consensus values for all six quality domains ranged from 31 to 100% and averaged 69%. The consensus values for the superordinate criterion ranged from 47 to 67%. No peer reported understanding or application problems dealing with the manual or the test items during the telephone interview. The only problems reported were due to the technical implementation but were solved.

### Inter-rater reliability of the peer review manual

Inter-rater reliability was evaluated by calculating average reliability coefficients for all reports that has been assessed by all peers. Overall, 325 reviews were conducted. Agreement on the superordinate criterion was highly heterogeneous and ranged from 29.2 to 87.5%. On average, agreement of all peers on the superordinate criterion was 47.3%. The corresponding inter-rater reliability value was r_w_ = 0.39. The reliability of the 22 test items of the subsidiary quality domains was heterogeneous with moderate variances. Depending on the item, reliability expressed as Kendall’s W ranged from 0.09 to 0.89 (Table 1). Coefficients of a discipline-specific evaluation (e.g., orthopaedic surgeons evaluating only orthopaedic reports, psychiatrists only psychiatric reports, etc.) showed similar characteristics as the main outcomes. Moreover, the selection of individual medical indications significantly reduced the number of cases included in the calculation. In fact, some coefficients could not be calculated or were based on very few cases. Regarding the superordinate criterion, surgery/orthopaedics had a lower (r_w_ = 0.18) and internal medicine/general practice had a slightly higher reliability value (r_w_ = 0.45) compared to the main evaluation outcome. The indication-specific reliability for neurology/psychiatry medical reports was r_w_ = 0.35.

### Peer judgment differences

**Fig. 1:**
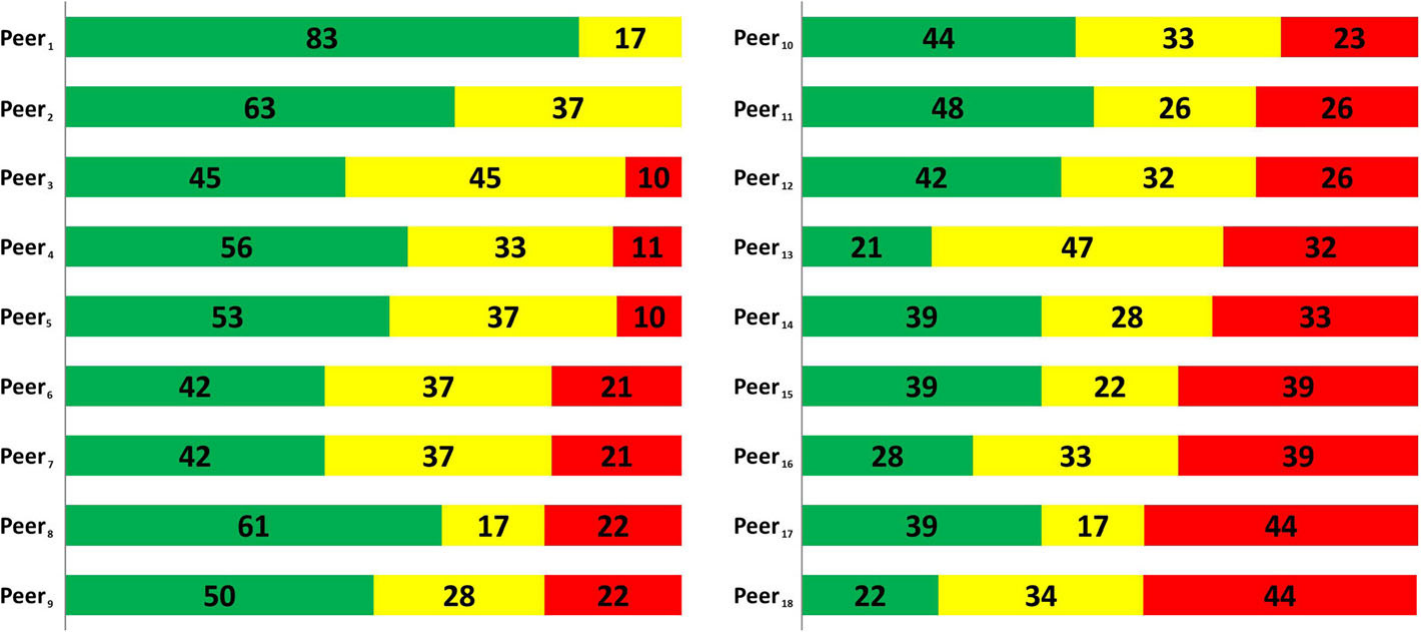
Percentage distribution of the superordinate criterion per peer. Figure shows peers judgements based on 20 medical reports reviewed by all peers for the superordinate criterion per peer. The calculation of percentage agreement demonstrate the individual usage of different deficiency categories when reviewing identical reports on a three-point rating scale; green colour: percentage of reports judged with no argument interruptions; yellow colour: percentage of reports judged with argument interruptions that can be bridged by the physician; red colour: percentage of reports judged with argument interruptions that cannot be bridged by the physician; *n* = 325 reviews; *n* = 18 peers

Considering that all peers judged the same reports there was a notable variance in peer assessments between the participating physicians concerning the superordinate criterion. The percentage distribution identified peer preferences for certain deficit scale severities. As seen in Fig. 1, individual peer bias exists within the review, illustrated here using line of argument. The most lenient peer_1_ assessed 83% of the reports to have no argument interruptions, whereas the strictest peer_18_ rated only 22% of these reports as adequate. Furthermore, two peers (peer_1_ and peer_2_) never assigned the highest scale category (argument interruptions that cannot be bridged by physicians) to any report.

## Discussion

### Interpretation of the inter-rater reliability

Our findings strongly support the expectation that peer reviews based on medical records rarely achieve common cut-off criteria for good inter-rater reliability. In general, a reliability value of 0.7 can be interpreted as good, yet our results suggest that the manual for quality assurance does not reach this level. Applying the common interpretation guidelines by Altman or Landis and Koch, two items have a slight, 17 items a fair, two items a moderate and one item a substantial reliability [34, 35]. With a value of 0.39, the superordinate criterion as the primary outcome demonstrated fair reliability. Analysis showed variations in reliability depending on the medical field of the peer. Internal medicine specialists and general practitioners showed the best agreement. A discipline-specific evaluation may not be recommended due to the small case quantity that was ultimately included in the reliability calculation. Besides the conventional interpretation standards we adapted our reliability interpretation based on similar research contexts as has been proposed in the literature [41]. For this purpose, the reliability (r_w_) of our main criterion was compared to peer review results from the external quality assurance programme in inpatient and outpatient medical rehabilitation in Germany [42]. Compared to this peer review, the reliability of the superordinate criterion is similar to the reliability coefficients of the peer review for the medical fields of orthopaedics (r_w_: 0.39 versus 0.35) and neurology/psychiatry (r_w_: 0.39 versus 0.30).

There are no graded interpretation guidelines for percentage agreement, as 100% indicates full and 0% indicated no consensus between two independent judges [43]. As a rule of thumb, percent agreement should be at least 75%, and good agreement can be defined as 90% or more [44]. Our range of agreement varies from 29.2 to 87.5% for test items of the subsidiary quality domains and reached 47.3% for the superordinate criterion. Thus, only four test items surpassed the minimum requirement.

Notably, the level of percentage agreement does not always correlate with inter-rater reliability. This reflects a statistical artefact on nominal or ordinal data. Reliability can be low if the majority of ratings are in the same deficit category, and thus not all categories are being exploited by the peers. The ratio of values influences reliability. Since reliability depends on how judgments are distributed, high reliability should not be expected if many ratings are in the same deficit category (see [44]). High percentage agreement based on one or two deficit categories indicates high concordance, but can lead to low reliability values. We have previously demonstrated that concordance and reliability of peer judgments do not occur by chance [38]. Considering these findings, results on percentage agreement can provide valid information, even if chance agreement is not taken into account.

### Pre-test reliability

During pre-test, no high significance values were found. However, the pre-test was not initially designed to achieve high reliability. The focus was on the elaboration of a comprehensive peer review manual and a factually-correct catalogue of test items. The calculation of reliability was performed to assess the feasibility of the peer review procedure. These rather low reliability values emphasised that objectivity and reliability can only be achieved if quality criteria were operationalized tightly. Accordingly, the manual and its rating scale were fundamentally revised. A further pre-test, which was not carried out, could have provided information whether these changes were sufficient.

### Confounding variables and sources of variance

A number of confounding factors may contribute to low reliability in a peer review process. According to our results, the main sources of variance seems to be the peer review instrument, the peer training, the peers and the medical experts’ reports itself. As previously explained, the lens models of social judgement theory outlines the basic phenomenon of different judgements [7]. Despite taking measures to ensure good reliability in constructing the peer review manual, we observed this systematic peer bias (Fig. 1). The unequal peer judgments have an impact on the magnitude of inter-rater reliability and may be the result of a systematic bias or from bias due to individual reviewer’s characteristics. Such bias, in which peers are systematically harsh or lenient, has been described in previous research and can be attributed to individuals’ differences such as gender, medical field, or other individual personal traits [45, 46].

Retrospectively, the systematic peer bias could have potentially been avoided if learning monitoring had been carried out during peer training. This would have enabled us to identify peers who did not use all levels of the ordinal rating scale. Such peers could have been specifically retrained to judge the test items according to the manuals’ instructions. Thus, while peer training was an integral part of our project, it was not sufficiently evaluated. Since peers did not provide any feedback regarding problems using the items, we proceeded to the evaluation phase. Future research should assess peer skills during training and intervene if necessary. Furthermore, targeted work in small groups with subsequent calibration may have its advantages, but we can’t be sure whether all peers have understood the application of the test item. The training was not standardized with predefined learning objectives and methods, and did not provide monitoring of trainees and moderators. Peer training should be more directed towards calibrating the peers to the rules of the manual.

The reliability reported in this study indicates that objectivity and reliability (as a precondition of validity) can only be achieved when: (1) the description of the items in the manual and their ordinal grading scale were tightly formulated, (2) peers who participating in the review process are provided with sufficient rater training, and (3) when the population (here: the medical experts’ reports) are sufficiently heterogeneous to allow distinction. Clear understandability of the different items is essential for uniform and unequivocal application.

During conception of the manual, a design with pre-defined rating scale anchor definitions for each item was chosen. A reason for limited reliability could be the four different types of anchor definition. In retrospect, it would have been more appropriate to use a uniform scale for assessing deficiencies. Even though each rating scale category was illustrated with examples, the peers reported occasional problems with their scale application. For example, problems were reported for the differentiation between socio-medically relevant and non-relevant measurement in test item C.3 or the quantitative differentiation between one, several and predominantly number of failures in test item B.2. To further increase reliability, the six subsidiary quality domains and the superordinate criterion could potentially be merged. In accordance with the typical structure of an evaluation score, the items scores could be summed and averaged instead of evaluating each item individually. This approach would make it possible to calculate the Intra Class Correlation (ICC) for interval scales [47], and to calculate Cronbach’s Alpha as a measure of internal consistency.

Another confounding variable is the medical report itself. The German Pension Insurance has articulated and published the requirements for socio-medical reports [39]. These requirements contain unified forms and provide specifications on the content and the formal structure of reports. The test items and quality domains in our peer review are based on these specifications and therefore measuring the quality of reports. Unfortunately, the validity of the report itself cannot be verified by the peer review. It is not possible to verify whether the expert, who wrote the report, followed the guideline. If the object of review itself is insufficient, reviewing it can be complicated and lead to inaccurate judgments.

Other confounding factors are worth noting. For example, a widely defined item like the superordinate criterion is harder to measure than a specific test item with predefined anchor definitions. Additionally, the number of scale categories may affect the level of concordance, as a low number of categories can reduce reliability coefficients. The distribution of peer assessments to the same deficit category on the quality rating scale can have a negative impact on the level of concordance. Nearly perfect agreement, without variance, can decrease reliability.

## Conclusion

Many studies have investigated the inter-rater reliability of peer review procedures and reported only fair to moderate reliability values [22–29]. Systematic development of a review tool has been recommended to counteract systematic bias. We attempted to construct a reliable peer review instrument taking into account recommendations from the literature. Although our instrument was pre-tested, revised and users have been trained, we merely achieved a fair inter-rater reliability in the main outcome (superordinate criterion). In summary, the reliability of our peer review manual was limited.

However, all participating physicians agreed that an approximation of peer judgments is possible through calibration to the rules of the manual among peers. In the context of our results, peers should receive standardized training before, and periodically after, the review process to improve reliability and to ensure fair quality comparisons. These regular training workshops would be effective if all peers involved in the quality assurance process are judging and discussing the same medical reports for which there are properly referenced reviews.

### Revision of the manual

The peer review manual was initially developed in the context of practical experience and preliminary considerations, which is a common procedure in the development of manuals. The iterative improvement was also based on the practical feedback from the pre-test. Nevertheless, the present study revealed that the manual requires further improvement. For practical application, the deficit categories with their four-point ordinal rating scales and anchor definitions were not always sufficiently distinguishing. The peers suggested that specific test items should be deleted for practical reasons. This suggestion, as well as low reliability scores, prompted us to remove five items from the latest version of the peer review manual (Table 1). The peers agreed that even a detailed description of the superordinate criterion would not genuinely enhance the evaluating process. At the same time, this criterion was considered as useful and relevant for judging a medical report. It has been rated as the most important and not interchangeable criterion for this quality assurance programme by the peers following the review phase. Using the superordinate criterion, the peers examined the link between different evaluation steps and the confident derivation of work capacity. Hence, this criterion judges the appropriate inner logic used to review a medical report. Proposed amendments to the superordinate criterion, however, were not sufficient, and therefore were not elaborated. Discussions with the peers revealed limitations in the specification of test items and our ordinal deficit grading system. These limitations do not originate exclusively in the manual itself, but also reflect the complexity of individual case constellations in evaluating the work capacity for disability pensions. The current version of the peer review manual can be retrieved from the homepage of the German Statutory Pension Insurance [48].

Previous literature [24, 31, 49] suggests that enhancing the peer review instrument seems to be a promising measure to reduce inter-rater variability. Improvement of the peer review manual and training of peers can enhance inter-rater reliability and reduces systematic bias [29, 31]. The peer review as an external quality assurance tool should not be applied in isolation; ideally, peer review should be complemented by measures of internal quality management. Our results suggest several desirable features for developing a valid and reliable peer review instrument: good and clear operationalisation of quality criteria, a refined and well evaluated manual, a standardized peer-training with adequate learning objectives and teaching methods for initial and continuing training, and sufficient opportunities for learning success control during and after training.

## Additional file

Additional file 1: **Table S1.** Subsidiary quality domain item D.3. Shows the structure of review item D.3 from the subsidiary quality domain completeness. The item consists of the test question, a detailed instruction on the rateable quality deficiencies and the four-point ordinal rating scale with pre-defined item-specific anchor definitions that describe possible quality restrictions. (DOCX 21 kb)

## Data Availability

The datasets analysed during the current study are available from the corresponding author on reasonable request.

## References

[1] Mumpower JL, Stewart TR (1996). Expert judgement and expert disagreement. Think Reason.

[2] Holdar U, Wallin L, Heiwe S (2013). Why do we do as we do? Factors influencing clinical reasoning and decision-making among physiotherapists in an acute setting. Physiother Res Int.

[3] Redelmeier DA, Tu JV, Schull MJ, Ferris LE, Hux JE (2001). Problems for clinical judgement: 2. Obtaining a reliable past medical history. CMAJ..

[4] Goldman RL (1994). The reliability of peer assessments. A meta-analysis. Eval Health Prof.

[5] Tait RC, Chibnall JT (1997). Physician judgments of chronic pain patients. Soc Sci Med.

[6] Chibnall JT, Dabney A, Tait RC, Cedraschi C, Nordin M, Nachemson A (2000). Internist judgments of chronic low back pain. Pain Med.

[7] Cooksey RW (1996). The methodology of social judgement theory. Think Reason.

[8] Wigton RS (1996). Social judgement theory and medical judgement. Think Reason.

[9] Way BB, Allen MH, Mumpower JL, Stewart TR, Banks SM (1998). Interrater agreement among psychiatrist in psychiatric emergency assessments. Am J Psychiatry.

[10] Götz M, Roth S, Chojetzki U (2011). Rechtliche Grundlagen für Leistungen der gesetzlichen Rentenversicherung. Sozialmedizinische Begutachtung für die gesetzliche Rentenversicherung.

[11] Vogel H, Zdrahal-Urbanek J (2004). Rehabilitation in Germany: new challenges of a structured social security scheme. Int J Rehabil Res.

[12] Cibis W, Hüller E (2003). Die sozialmedizinische Begutachtung. Sozialmedizinische Begutachtung für die gesetzliche Rentenversicherung.

[13] Legner R, Cibis W (2007). Quality assurance in sociomedical evaluation. Rehabilitation..

[14] Cibis W, Paulus E-M, Mai H, Gehrke J (2011). Die sozialmedizinische Begutachtung. Sozialmedizinische Begutachtung für die gesetzliche Rentenversicherung.

[15] Ueberschär I (2008). Quality assurance in the socio-medical assessment in the German pension insurance. Gesundheitswesen..

[16] Goldman RL (1992). The reliability of peer assessments of quality of care. JAMA..

[17] Shaw C (2001). External assessment of health care. BMJ..

[18] Jäckel WH, Farin E (2004). Quality assurance in rehabilitation: where do we stand today?. Rehabilitation..

[19] Kottner J, Audigé L, Brorson S, Donner A, Gajewski BJ, Hróbjartsson A (2011). Guidelines for reporting reliability and agreement studies (GRRAS) were proposed. J Clin Epidemiol.

[20] Wirtz M, Caspar F (2002). Beurteilerübereinstimmung und Beurteilerreliabilität.

[21] Broder MS, Oken C, Parker M, Giammona M, Newman J, Charlene H (2002). Outpatient care: a conceptual framework and a form for structured implicit review.

[22] Abragam A, Lincke H-O, Lux A, Wallesch CW (2002). Peer review of routine clinical case reports - an instrument of quality management? Results of a pilot investigation. Nervenarzt..

[23] Dharmar M, Marcin JP, Kuppermann N, Andrada ER, Cole S, Harvey DJ (2007). A new implicit review instrument for measuring quality of care delivered to pediatric patients in the emergency department. BMC Emerg Med.

[24] Harris CD, Bratzler DW (2013). Evaluating quality of care: the role of peer review. J Okla State Med Assoc.

[25] Hayward RA, Bernard AM, Rosevear JS, Anderson JE, McMahon LF (1993). An evaluation of generic screens for poor quality of hospital care on a general medicine service. Med Care.

[26] Kadar N (2010). Systemic bias in peer review: suggested causes, potential remedies. J Laparoendosc Adv Surg Tech A.

[27] Kameoka J, Okubo T, Koguma E, Takahashi F, Ishii S, Kanatsuka H (2014). Development of a peer review system using patient records for outcome evaluation of medical education: reliability analysis. Tohoku J Exp Med.

[28] Neuderth S (2004). Externe Qualitätssicherung durch peer-review.

[29] Smith MA, Atherly AJ, Kane RL, Pacala JT (1997). Peer review of the quality of care. JAMA..

[30] Lilford R, Edwards A, Girling A, Hofer T, Di Tanna GL, Petty J (2007). Inter-rater reliability of case-note audit: a systematic review. J Health Serv Res Policy.

[31] Tuijn S, Janssens F, Robben P, van den Bergh H (2012). Reducing interrater variability and improving health care: a meta-analytical review. J Eval Clin Pract.

[32] de Vet HCW, Mokkink LB, Terwee CB, Hoekstra OS, Knol DL (2013). Clinicians are right not to like Cohen’s κ. BMJ..

[33] Wirtz M, Kutschmann M (2007). Analyzing interrater agreement for categorical data using Cohen’s kappa and alternative coefficients. Rehabilitation..

[34] Altman DG. Practical statistics for medical research. London: Chapman and Hall/CRC; 1991.

[35] Landis JR, Koch GG (1977). The measurement of observer agreement for categorical data. Biometrics..

[36] Deutsche Rentenversicherung Bund (2009). Qualitätssicherung der sozialmedizinischen Begutachtung: Bericht zur Umsetzun des “Qualitätssicherungsverfahrens der sozialmedizinischen Begutachtung” - Aktueller stand.

[37] Bortz J, Lienert GA, Boehnke K (2008). Verteilungsfreie Methoden in der Biostatistik.

[38] Strahl A, Gerlich C, Wolf H-D, Gehrke J, Müller-Garnn A, Vogel H (2016). Quality Assurance in Sociomedical Evaluation by peer review: a pilot project of the German statutory pension insurance. Gesundheitswesen..

[39] Verband Deutscher Rentenversicherungsträger (VDR). DRV-Schriften, Band 21: Das ärztliche Gutachten für die gesetzliche Rentenversicherung. Frankfurt/Main: Verband Deutscher Rentenversicherungsträger; 2001.

[40] Ethik-Kommission der Bayerischen Landesärztekammer (EK) (2014). Antrag an die Ethik-Kommission (EK) auf Beurteilung eines Forschungsvorhabens am menschen, das nicht unter die Anforderungen der §§ 40 ff AMG btw. 20 ff MPG fällt. EK.

[41] Goulet F, Jacques A, Gagnon R, Racette P, Sieber W (2007). Assessment of family physicians’ performance using patient charts: interrater reliability and concordance with chart-stimulated recall interview. Eval Health Prof.

[42] Farin E, Carl C, Lichtenberg S, Jäckel WH, Maier-Riehle B, Rütten-Köppel E (2003). Evaluating the rehabilitation process by means of peer review: examination of the methods used and findings of the 2000/2001 data collection in the somatic indications. Rehabilitation..

[43] Allen M (2017). The SAGE encyclopedia of communication research methods: volume 1.

[44] Shweta BRC, Chaturvedi HK (2015). Evaluation of inter-rater agreement and inter-rater reliability for observational data: an overview of concepts and methods. J Indian Acad Appl Psychol.

[45] Hulka BS, Romm FJ, Parkerson GR, Russell IT, Clapp NE, Johnson FS (1979). Peer review in ambulatory care: use of explicit criteria and implicit judgments. Med Care.

[46] Lee CJ, Sugimoto CR, Zhang G, Cronin B (2013). Bias in peer review. J Am Soc Inf Sci Technol.

[47] Graham M, Milanowski A, Miller J, (Center for Education Compensation and Reform) (2012). Measuring and promoting inter-rater agreement of teacher and principal performance ratings.

[48] Deutsche Rentenversicherung Bund. Qualitätssicherung der sozialmedizinischen Begutachtung: manual zum peer review-Verfahren: DRV; 2018. Available from: https://www.deutsche-rentenversicherung.de/SharedDocs/Downloads/DE/Experten/infos_fuer_aerzte/begutachtung/manual_peer_review.pdf?__blob=publicationFile&v=1.

[49] Hofer TP, Asch SM, Hayward RA, Rubenstein LV, Hogan MM, Adams J (2004). Profiling quality of care: is there a role for peer review?. BMC Health Serv Res.

