# Supplementary material for: An instrument for quality assurance in work capacity evaluation: development, evaluation, and inter-rater reliability
Source: BMC Health Serv Res. 2019 Aug 9;19:556. doi: 10.1186/s12913-019-4387-4 (PMC6688267; doi:10.1186/s12913-019-4387-4)
Supplement: Supplementary file 1 — Table S1. Subsidiary quality domain item D.3. Shows the structure of review item D.3 from the subsidiary quality domain completeness. The item consists of the test question, a detailed instruction on the rateable quality deficiencies and the four-point ordinal rating scale with pre-defined item-specific anchor definitions that describe possible quality restrictions. (DOCX 21 kb) [file 12913_2019_4387_MOESM1_ESM.docx]

**Additional file 1: Table S1: subsidiary quality domain item D.3**

| **Quality domain: completeness Review item D.3** |
| --- |
| **D.3 To what extent are ICD diagnoses illustrated with their functional limitations?**  According to the specifications of the DRV script volume 21 “The medical experts’ report for the German Statutory Pension Insurance”:   - Diagnoses must be formulated in writing (e.g., bronchial asthma) and encrypted according to the valid ICD Code - Diagnoses must be listed in the free text of the report; the first three social-medical relevant diagnoses must be included in the back banner page of the unified form - Diagnoses must be illustrated with their functional limitations (e.g., drug-adjusted diabetes mellitus type II without significant consequential damage, e.g., chronic obstructive pulmonary disease with permanent severe respiratory impairment) - Diagnoses should be arranged according to the severity of their functional limitations, with classification and/or staging, if applicable.   The ICD encryption is required, if possible, with four digits (including the digit after the dot, e.g., J45.1). The encryption on the back banner page from the unified form is sufficient for this.  The extent to which a diagnosis can be derived from the anamnesis or clinical examination should not be evaluated here. It is also not possible to evaluate whether the diagnosis is valid. |

| Mild deficiencies | At least one of the first three diagnoses has no ICD encryption. |
| --- | --- |
| Clear deficiencies | The linguistic formulation of the diagnoses (e.g., bronchial asthma) is missing.  OR  The function limitations are not specified. |
| Serious deficiencies | The linguistic formulation of the diagnoses (e.g., bronchial asthma) is missing.  AND  The functional limitations are not specified. |

| **Which deficiencies** **were identified?**  The judgement shall be based exclusively on the content of the test item and shall be carried out in the specified deficiencies categories. If none of the three deficiencies categories is applicable, the category “no deficiency” shall be coded. The quality deficits on which the peer judgment is based must be documented. |
| --- |
